# The role of FDG PET assessment in patients with advanced breast cancer treated with cyclin-dependent kinase 4/6 inhibitors in the second-line setting

**DOI:** 10.3389/fonc.2024.1454844

**Published:** 2024-12-03

**Authors:** Marcin Kubeczko, Anna Polakiewicz-Gilowska, Andrea D’Amico, Olgierd Chrabański, Katarzyna Świderska, Ewa Chmielik, Sławomir Blamek, Daria Handkiewicz-Junak, Michał Jarząb

**Affiliations:** ^1^ Breast Cancer Center, Maria Sklodowska-Curie National Research Institute of Oncology, Gliwice, Poland; ^2^ Department of Nuclear Medicine and Endocrine Oncology, Maria Sklodowska-Curie National Research Institute of Oncology, Gliwice, Poland; ^3^ Tumor Pathology Department, Maria Sklodowska-Curie National Research Institute of Oncology, Gliwice, Poland; ^4^ Department of Radiotherapy, Maria Sklodowska-Curie National Research Institute of Oncology, Gliwice, Poland

**Keywords:** advanced breast cancer, 18Ffluorodeoxyglucose positron emission tomography, palbociclib, ribociclib, abemaciclib

## Abstract

**Background:**

Cyclin-dependent kinase 4/6 (CDK4/6) inhibitors have demonstrated a survival benefit in the second-line treatment of patients with hormone receptor-positive human epidermal growth factor receptor 2-negative advanced breast cancer. However, identifying prognostic biomarkers remains a challenge. Thus, we aimed to assess the prognostic value of 18F-fluorodeoxyglucose positron emission tomography-computed tomography (FDG-PET-CT) performed before CDK4/6 inhibitors initiation.

**Methods:**

This single-center retrospective analysis comprised patients treated with CDK4/6 inhibitors in the second-line setting between 2018 and 2024, with FDG-PET-CT performed before CDK4/6 inhibitor initiation.

**Results:**

The study included 39 patients with a median age of 63 years (IQR 50 -71). Among them, 12 had *de novo* metastatic disease (30.8%), and 13 had oligometastatic disease (33.3%). Treatment distribution was as follows: 15 patients received palbociclib (38%), 19 ribociclib (49%), and five abemaciclib (13%). Most patients received fulvestrant (31 patients, 79%), whereas eight patients (21%) were treated with letrozole. The median progression-free survival (PFS) in all studied patients was 25.8 months. Notably, baseline SUVmax (maximum standardized uptake value) showed statistically and clinically significant differences. Patients in the low SUVmax group had a median PFS of 30.7 months, compared to 13.0 months for those in the high SUVmax group (p = 0.038). The 2-year PFS was 76.2% [95% CI 51.8% - 89.4%] for the low SUVmax group, contrasting with 22.3% [95% CI 4.0% - 49.9%] for the high SUVmax group. High SUVmax, poor performance status, and *de novo* metastatic disease were independent prognostic factors for PFS.

**Conclusions:**

FDG-PET-CT performed before cyclin-dependent kinase 4/6 inhibitor commencement is a valuable prognostic tool in hormone receptor-positive human epidermal growth factor receptor 2-negative advanced breast cancer. Patients with SUVmax less than 8.4 experienced extended progression-free survival compared to those with higher SUVmax.

## Introduction

Cyclin-dependent kinase 4/6 inhibitors, in combination with endocrine therapy, represent the gold standard for treating patients diagnosed with hormone receptor-positive human epidermal growth factor 2-negative metastatic breast cancer (1, 2). Numerous studies have confirmed the efficacy of CDK4/6 inhibitors in patients who have progressed while receiving endocrine therapy (3–5), resulting in a significant progression-free survival advantage (4). Additionally, some studies have observed an overall survival benefit with this combination (6, 7).

Current guidelines recommend CDK4/6 inhibitors as either first-line or second-line treatment for patients with advanced breast cancer (1, 2). Typically, most patients receive this combination in the first-line setting. However, the SONIA trial raised questions about the optimal position for CDK4/6 inhibitors (8). Surprisingly, first-line use of CDK4/6 inhibitors in combination with endocrine therapy did not yield statistically significant or clinically meaningful progression-free survival benefits compared to second-line treatment (9). Moreover, due to prolonged exposure to CDK4/6 inhibitors in the first line, increased toxicity and higher costs were observed, prompting consideration of second-line use as a more favorable option for most patients (9).

On the other hand, the addition of inavolisib to palbociclib and fulvestrant in the first line improved progression-free survival (10). Thus, biomarkers predicting the duration of response might select patients for escalation or de-escalation strategy. Imaging studies remain a fundamental tool for monitoring treatment efficacy since resistance to CDK4/6i occurs frequently (11). While liquid biopsy using ctDNA (12) or miRNA (13) shows promise in monitoring treatment resistance, imaging remains the standard of care (1). Assessment of response to treatment is commonly performed using the Response Evaluation Criteria in Solid Tumors (14). In pivotal trials related to the use of CDK4/6 inhibitors in combination with endocrine therapy for metastatic breast cancer, imaging studies were conducted every 6-12 weeks (3–5). However, in clinical practice, a reasonable frequency for routine monitoring of advanced breast cancer patients is suggested to be every two to four months (1).

Biomarkers, which could help to stratify patients between long-responders and early progressors, are urgently needed (15). Positron emission tomography (PET) with the 18F-fluorodeoxyglucose (FDG) radiotracer is a valuable tool in breast cancer management, particularly for staging and evaluating equivocal findings from other imaging modalities (16). Metabolic parameters obtained from PET scans may provide additional insights (17). Several studies have demonstrated that FDG-PET-computed tomography (CT) may offer superior sensitivity and prognostic value compared to contrast-enhanced CT (CE-CT) in monitoring treatment response in metastatic breast cancer, particularly in detecting regressive disease and bone metastases (18–21).

However, data on the utility of PET-CT in patients undergoing CDK4/6 inhibitor treatment are limited, especially in the second-line setting (22–26). Unfortunately, due to the low number of patients included, no specific data exists for patients treated with CDK4/6 inhibitors exclusively in the second-line setting.

In this study, we aimed to assess the role of baseline PET-CT assessment in patients with advanced breast cancer who received CDK4/6 inhibitors in second-line setting.

## Methods

### Studied population

The study focused on patients diagnosed with advanced hormone receptor-positive human epidermal growth factor receptor 2-negative breast cancer. These patients received treatment with cyclin-dependent kinase 4/6 (CDK4/6) inhibitors in combination with endocrine therapy as second-line treatment. All patients underwent 18F-fluorodeoxyglucose positron emission tomography-computed tomography before CDK4/6 inhibitors commencement. This single-center retrospective analysis included patients treated between 2018 and 2024.

The primary endpoint of the study was to assess the association between the maximum standardized uptake value (SUVmax) measured using FDG-PET-CT and progression-free survival during CDK4/6 inhibitors treatment.

### Imaging

18F-fluorodeoxyglucose positron emission tomography-computed tomography (PET-CT) scans were performed using either the Gemini XL (Philips Medical Systems, Eindhoven, the Netherlands) or the Siemens Biograph mCT (Siemens Healthineers, Erlangen, Germany), following the internal protocol, including intravenous infusion of the 18F-FDG (3.7MBq/kg, with activities ranging between 185 and 555 MBq) after 6 hours of fasting. Scanners underwent calibration to measure the patient-prepared activity accurately. The acquisition process commenced 60 minutes after the injection of the radiotracer. The highest standardized uptake value (SUVmax) within the volume of interest, extending from the skull base to the mid-thigh level, was used as the primary metabolic parameter in our study.

### Response assessment

To monitor treatment efficacy, contrast-enhanced computed tomography scans were performed at baseline and every three months thereafter. Response to treatment was assessed using the Response Evaluation Criteria in Solid Tumors version 1.1 criteria (RECIST v. 1.1). The evaluation categorized responses as follows: complete response (CR), partial response (PR), progressive disease (PD), or stable disease (SD) (14). Progression-free survival was calculated from the date of CDK4/6 inhibitor initiation to the date of disease progression or death, whichever occurred first. Overall survival was calculated from the date of CDK4/6 inhibitor initiation to the date of death.

### Statistical analyses

Differences between categorical variables were assessed using the Fisher exact test, whereas differences between continuous variables were assessed using the Wilcoxon rank sum test. Survival analysis was performed using the Kaplan-Meier method. Differences in survival were evaluated using the log-rank test. Variables with a p-value less than 0.2 in the univariate Cox regression were included in the multivariate Cox regression. All tests were two-sided, and statistical significance was set at a p-value less than 0.05. The analyses were conducted using Stata Statistical software (version 18, StataCorp, College Station, TX, USA).

### Determination of the optimal SUVmax cutpoint

Recent key phase III clinical trials evaluating CDK4/6 inhibitors in combination with endocrine therapy have demonstrated significantly prolonged PFS compared to endocrine therapy alone. In the MONALEESA-3 trial, patients in the ribociclib arm achieved a median PFS of 20.5 months (5), while in the MONALEESA-2 trial, the median PFS was 25.3 months (27). For abemaciclib, the median PFS was 28.2 months in the MONARCH 3 trial (28) and 16.4 months in the MONARCH 2 trial (3). Palbociclib, in the PALOMA-2 trial, demonstrated a median PFS of 25.3 months (29). Based on these findings, we selected 24-month PFS as the reference point for determining the optimal SUVmax cutpoint. The cutpoint was determined using the Receiver Operator Characteristics curve and the Youden index. The optimal cutpoint for SUVmax was identified as 8.4, with a sensitivity of 0.67, a specificity of 0.85, and area under the ROC curve (AUC) of 0.76 at this threshold. The Receiver Operating Characteristic (ROC) curve, along with the corresponding Area Under the Curve (AUC) for SUVmax, is displayed in **Supplementary Figure 1**.

## Results

### Patients characteristics

Thirty-nine patients treated with cyclin-dependent kinase 4/6 (CDK4/6) inhibitors in the second-line setting with FDG-PET/CT performed before CDK4/6 inhibitor initiation were included in the analysis. The median age was 63 years, with the interquartile range (IQR) of 50 – 71 years. Twelve patients had *de novo* metastatic disease (30.8%), while twenty-seven patients were diagnosed with recurrent disease (69.2%). Thirteen patients (33.3%) had oligometastatic disease. Sixteen patients had a performance status of 0 (41%) according to Eastern Cooperative Oncology Group (ECOG), eighteen patients ECOG 1 (46%), and five patients ECOG 2 (13%). The majority of patients had luminal B HER2-negative breast cancer intrinsic subtype (twenty-seven patients, 69%), while eight patients had luminal A intrinsic subtype (eight patients, 21%). In four patients, the intrinsic subtype was not assessed due to the lack of Ki67 assessment. Liver metastases were present in nine patients, whereas lung metastases in fourteen patients. Fifteen patients received palbociclib (38%), nineteen ribociclib (49%), and five abemaciclib (13%). Regarding endocrine therapy, the vast majority of patients received fulvestrant (31 patients, 79%), whereas eight patients (21%) were treated with letrozole. The median interval between PET-CT and CDK4/6 inhibitor commencement was 1.4 months.

The median follow-up from CDK4/6 inhibitor treatment commencement was 16.9 months (IQR 10.4 – 32.6 months). CDK4/6 inhibitors dose required modification due to toxicity in eighteen patients. Specifically in fourteen patients (60.9%) in the low SUVmax group and four patients (25.0%) in the high SUVmax group (p = 0.049).

### 18F-fluorodeoxyglucose positron emission tomography – computed tomography assessment

**Figure 1:**
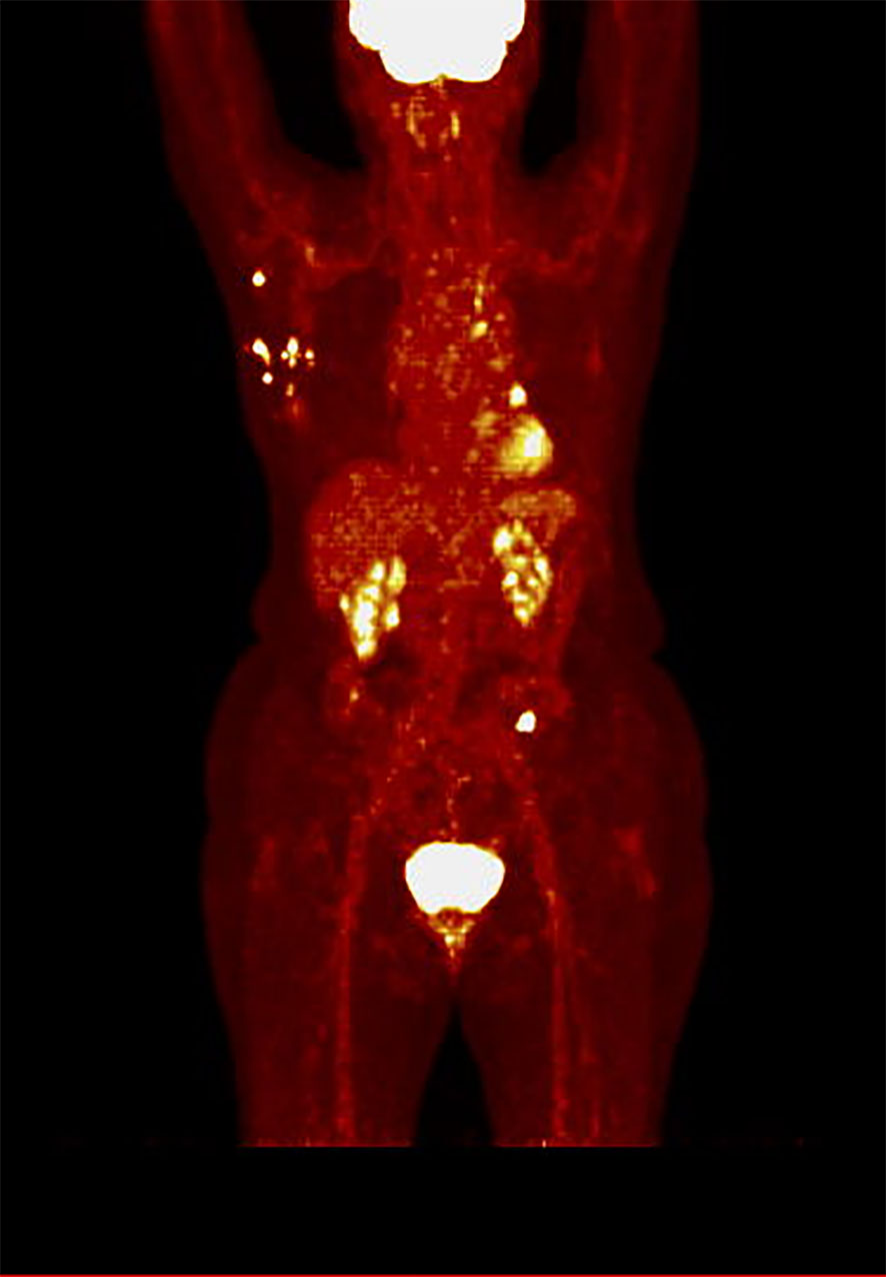
PET scan in hot iron color scale of a patient from the low SUVmax group (SUVmax 7.8), demonstrating multiple visceral and bone metastases, including metastases to the liver, lungs, pleura, bones, and lymph nodes.

**Figure 2:**
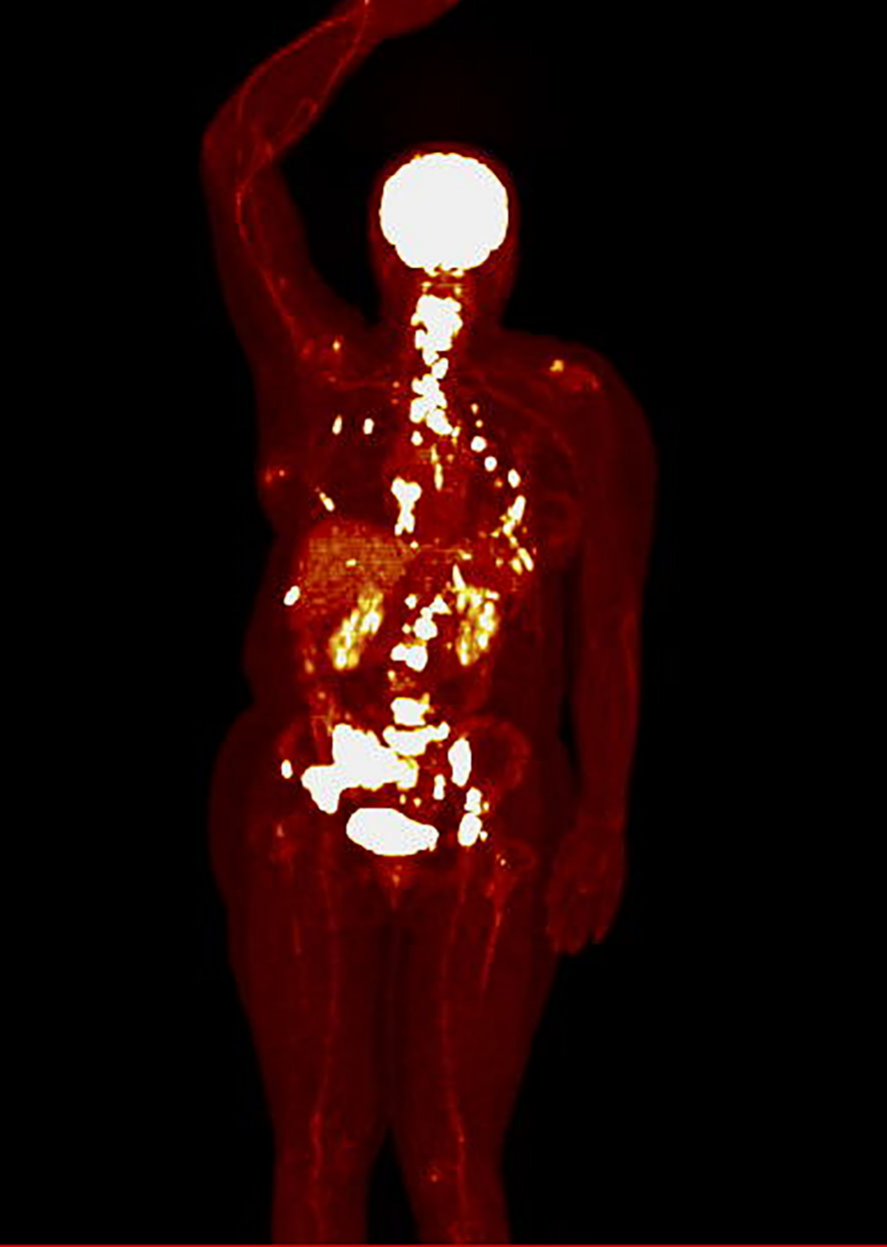
PET scan in hot iron color scale of a patient from the high SUVmax group (SUVmax 24.4), showing primarily bone metastases, along with metastases to the pleura and internal mammary lymph nodes.

The mean SUVmax was 8.4 [SD ± 4.9], ranging from 2.4 to 24.4. Twenty-three patients had SUVmax < 8.4 before CDK4/6 inhibitors initiation (defined as low SUVmax, while sixteen patients had SUVmax ≥ 8.4 (defined as high SUVmax). An example of a PET scan image from a patient in the low SUVmax group is presented in **Figure 1**, while from a patient in the high SUVmax group is shown in **Figure 2**.

A comparison between patients in the low SUVmax group and the high SUVmax group is depicted in **Table 1**.

**Table 1:** Comparison between patients in low SUVmax group and high SUVmax group.

Characteristics	Low SUVmax [n=23]	High SUVmax [n=16]	p
**Age**, median [IQR]	64 [60 – 71]	58.5 [47 – 67]	0.209
ECOG performance status			
ECOG 0	9 [39.1%]	7 [43.8%]	0.743
ECOG 1	10 [43.5%]	8 [50.0%]	
ECOG 2	4 [17.4%]	1 [6.2%]	
***De novo* metastatic**	7 [33.3%]	5 [27.8%]	0.742
**Oligometastatic disease**	7 [30.4%]	6 [37.5%]	0.736
**Luminal B** HER2-negative intrinsic subtype	14 [60.9%]	13 [81.3%]	0.512
**Bsl liver metastases**	4 [17.4%]	5 [31.3%]	0.444
**Bsl lung metastases**	7 [30.4%]	7 [43.8%]	0.503
CDK4/6 inhibitor			
Palbociclib	9 [39.1%]	6 [37.5%]	0.208
Ribociclib	13 [56.5%]	6 [37.5%]	
Abemaciclib	1 [4.4%]	4 [25.0%]	
Endocrine Tx			
Letrozole	6 [26.1%]	2 [12.5%]	0.432
Fulvestrant	17 [73.9%]	14 [87.5%]	

IQR, interquartile range; ECOG, performance status according to Eastern Cooperative Oncology Group; Bsl, baseline; CDK4/6 inhibitor, cyclin-dependent kinase 4/6 inhibitor; Tx, treatment.

### Treatment efficacy

**Figure 3:**
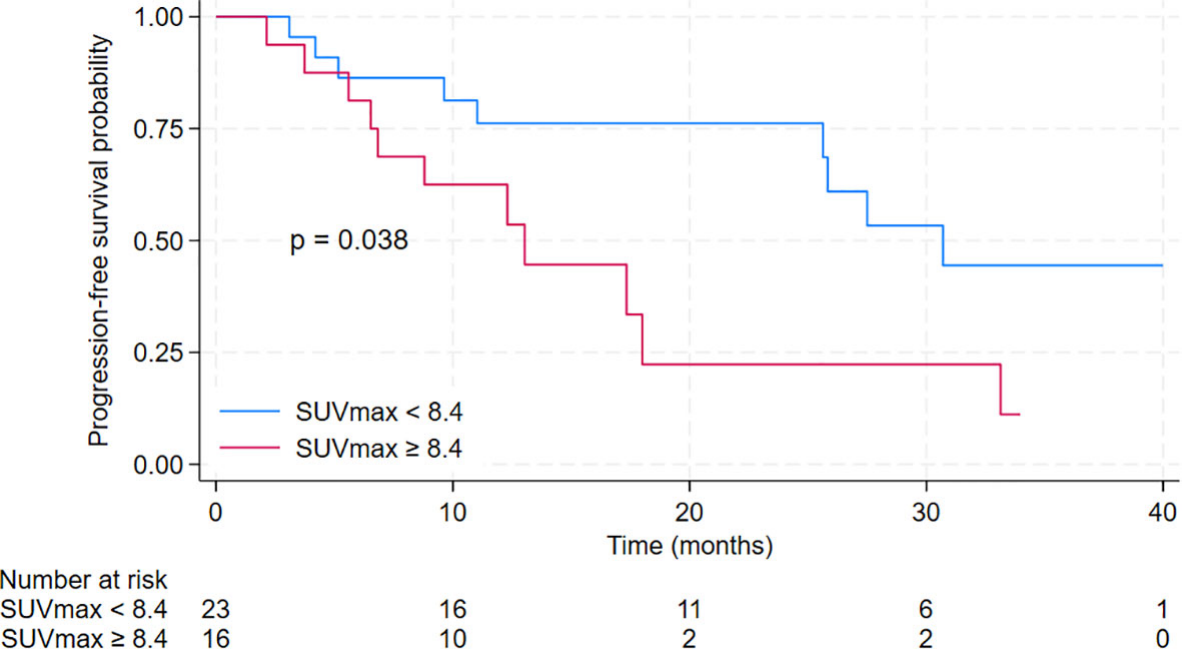
Kaplan-Meier curves for progression-free survival of patients treated with cyclin-dependent kinase 4/6 inhibitors in the second-line setting with a comparison between low SUVmax group (defined as baseline SUVmax <8.4) and high SUVmax group (defined as baseline SUVmax ≥ 8.4).

**Figure 4:**
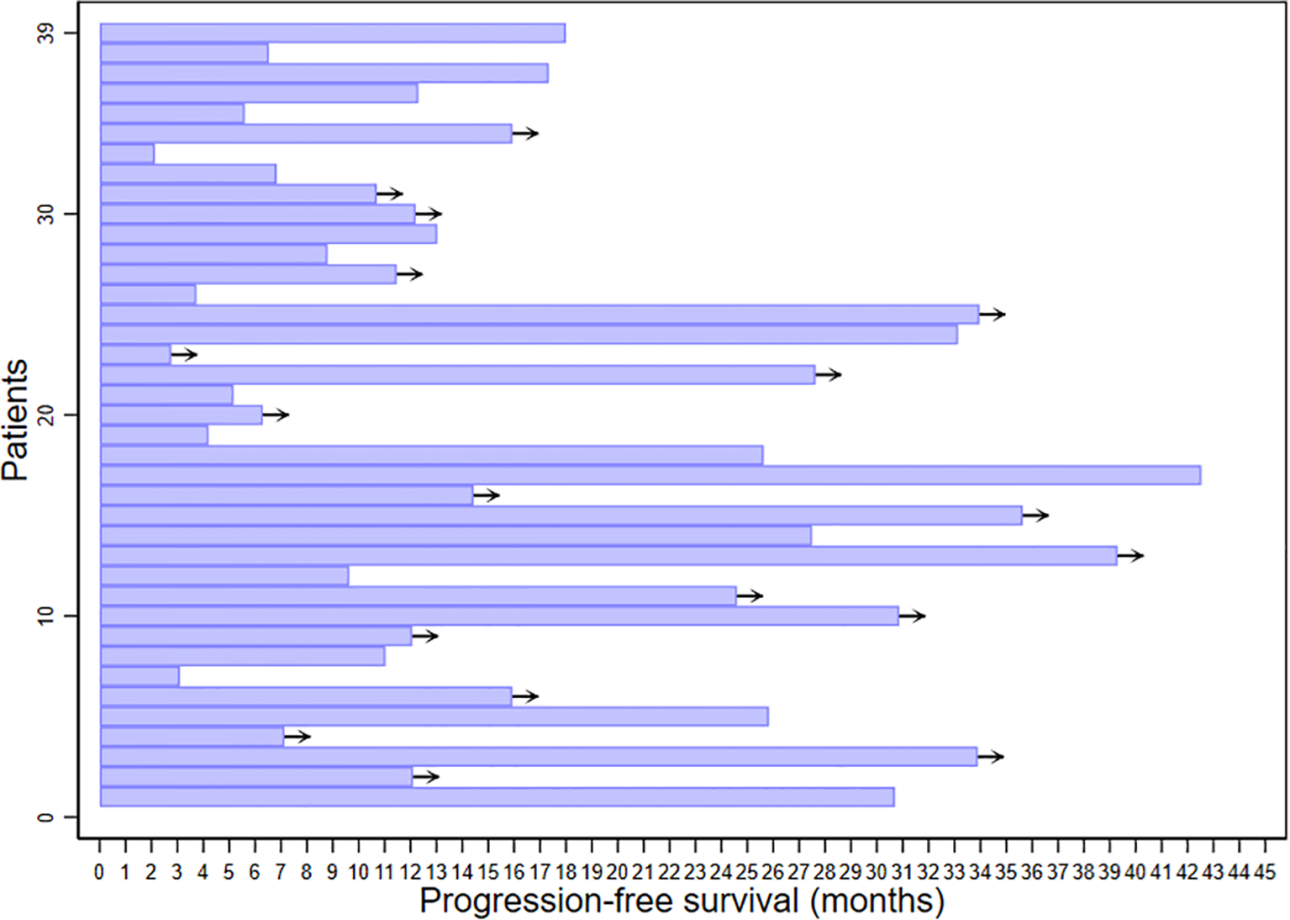
Duration of response to cyclin-dependent kinase 4/6 inhibitors therapy among studied patients. Arrows indicate ongoing treatment.

The median progression-free survival (PFS) for all studied patients was 25.8 months [95% CI 12.3 – 33.1 months]. There were statistically and clinically significant differences according to baseline SUVmax. The median PFS for patients in the low SUVmax group was 30.7 months [95% CI 25.6 months – not reached], compared to 13.0 months [95% CI 6.5 – 18 months] for patients in the high SUVmax group (p = 0.038). The 2-year PFS was 76.2% [95% CI 51.8% - 89.4%] for the low SUVmax group compared to only 22.3% [95% CI 4.0% - 49.9%] for the high SUVmax group. PFS results are displayed in **Figure 3**. The duration of response is illustrated in **Figure 4**.

**Figure 5:**
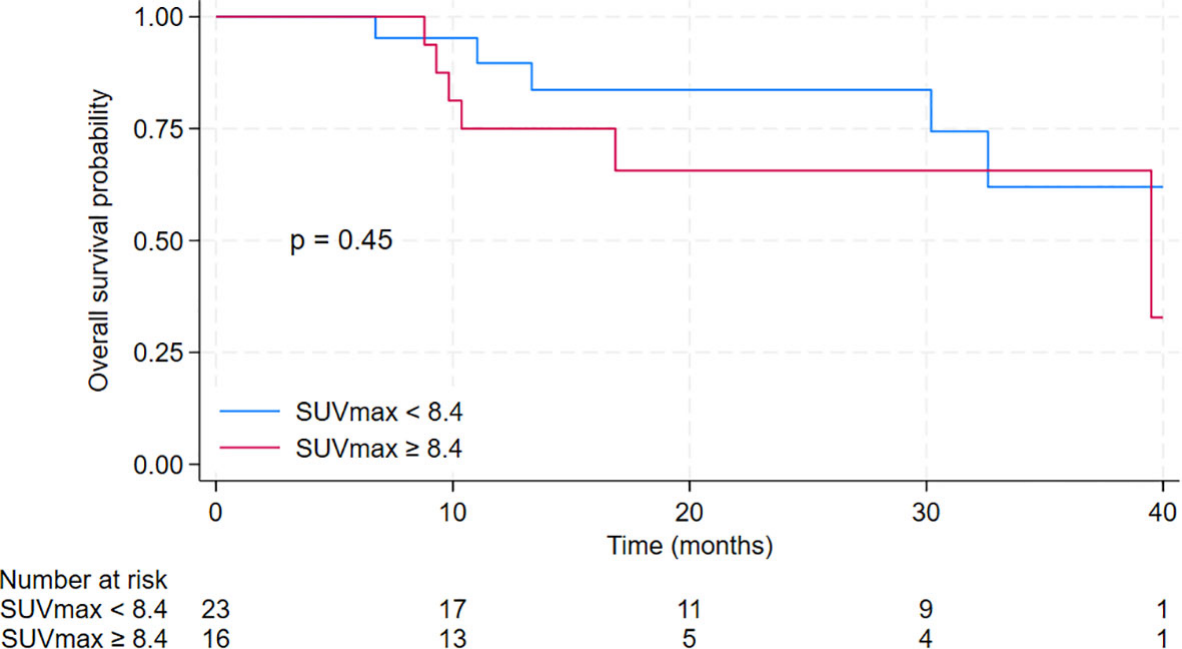
Kaplan-Meier curves for overall survival of patients treated with cyclin-dependent kinase 4/6 inhibitors in the second-line setting with a comparison between low SUVmax group (defined as baseline SUVmax <8.4) and high SUVmax group (defined as baseline SUVmax ≥ 8.4).

The median overall survival (OS) for the entire cohort was 39.5 months [95% CI 30.2 months – not reached]. The median OS for patients in the low SUVmax group was not reached [95% CI 30.2 months – not reached], compared to 39.5 months [95% CI 10.4 months – not reached] for patients in the high SUVmax group. The 2-year OS was 83.7% [95% CI 57.2% - 94.5%] for the low SUVmax group compared to 65.6% [95% CI 34.9% - 84.5%] for the high SUVmax group. However, the difference was not significant (p = 0.45). Results are displayed in **Figure 5**.

At the data cut-off, twenty-one patients experienced disease progression while on CDK4/6 inhibitor therapy. All patients eligible for further treatment received chemotherapy, with the following distribution: seven patients were treated with capecitabine, five with paclitaxel, one with non-pegylated liposomal doxorubicin, and one with a combination of capecitabine and fulvestrant. Seven patients did not receive subsequent therapy, primarily due to a decline in performance status.

Poorer performance status and high SUVmax were associated with an increased risk of progression. These associations were observed in both univariate analysis and multivariate Cox regression. For detailed results, please refer to **Table 2**.

**Table 2:** Cox proportional regression model for progression-free survival.

Variable	HR [95% CI; p]
Univariate analysis	
Age [continuous]	0.97 [95% CI 0.93 – 1.00; p = 0.057]
ECOG 2 *vs*. ECOG 0/1	1.80 [95% CI 1.07 – 3.02; p = **0.026**]
*De novo* metastatic *vs*. recurrent disease	0.39 [95% CI 0.11 – 1.32; p = 0.128]
Oligometastatic *vs*. polymetastatic disease	0.71 [95% CI 0.26 – 1.96; p = 0.510]
Liver metastases *vs*. no liver metastases	1.11 [95% CI 0.37 – 3.34; p = 0.853]
SUVmax ≥ 8.4 *vs*. SUVmax < 8.4	2.52 [95% CI 1.03 – 6.21; p = **0.044**]
Multivariate analysis	
Age [continuous]	0.97 [95% CI 0.93 – 1.00; p = 0.081]
ECOG 2 *vs*. ECOG 0/1	5.9 [95% CI 1.78 – 19.8; p = **0.004**]
*De novo* metastatic *vs*. recurrent disease	0.22 [95% CI 0.06 – 0.84; p = **0.026**]
SUVmax ≥ 8.4 *vs*. SUVmax < 8.4	3.80 [95% CI 1.27 – 11.39; p = **0.017**]

Statistically significant results are highlighted in bold.

### Histopathological subtypes and SUVmax

Of the patients included in the study, thirty-three had invasive carcinoma of no special type (NST), five had invasive lobular carcinoma (ILC), and one patient’s histopathological subtype was missing due to incomplete records. This missing data occurred in a patient who had been treated for early breast cancer more than a decade ago and experienced disease recurrence, but unfortunately, details regarding the histopathological subtype were unavailable.

All five patients with ILC were in the low SUVmax group. The median SUVmax for patients with ILC was 4.7 [IQR 4.7–5.4], while for those with NST, the median SUVmax was 8.1 [IQR 4.9–11.9]. Despite these numerical differences, the differences were not statistically significant (p = 0.08). Additionally, we found no significant difference in PFS (p = 0.89) or OS (p = 0.24) between patients with ILC and NST histopathology. The 2-year PFS was 55.9% [95% CI 34.5%–72.8%] for NST and 60.0% [95% CI 12.6%–88.2%] for ILC. The 2-year OS was 81.6% [95% CI 60.8%–92.0%] for NST compared to 53.3% [95% CI 6.8%–86.3%] for ILC.

## Discussion

Cyclin-dependent kinase 4/6 inhibitors are effective as a second-line treatment for hormone receptor-positive human epidermal growth factor 2-negative advanced breast cancer. However, identifying reliable biomarkers for predicting treatment response remains an unmet need. Our study investigated the utility of positron emission tomography-computed tomography (PET-CT) as a prognostic tool in this patient population. Notably, we found that the maximum standardized uptake value (SUVmax) on PET-CT scans emerged as a strong independent prognostic factor associated with progression-free survival (PFS). Results are also clinically meaningful: the median PFS for patients with low baseline SUVmax, defined as SUVmax less than 8.4, was more than two times longer than in patients with high baseline SUVmax, specifically 30.7 months versus 13.0 months. Despite a higher rate of CDK4/6 inhibitor dose modifications due to toxicity, the low SUVmax group demonstrated better survival outcomes.

Additionally, poor performance status and recurrent disease (as opposed to *de novo* metastatic disease) were consistent negative prognostic factors, aligning with previous research (30, 31). The overall survival observed in our study was 39.5 months, closely resembling results from the PALOMA-3 (39.7 months) (4) and MONALEESA-3 (40.2 months) trials (7).

Prognostic biomarkers for patients with advanced breast cancer are urgently needed, particularly as new frontline treatment regimens emerge (10). In this context, our study sheds light on promising approaches for predicting response and optimizing patient outcomes.

The number of patients eligible for second-line CDK4/6 inhibitors is undoubtedly small when considering that the majority of patients receive CDK4/6 inhibitor as a first-line treatment in combination with endocrine therapy. However, in a large population of patients with metastatic ER+/HER2-negative breast cancer, even a small proportion of patients being treated with second-line CDK4/6 inhibitors still results in a substantial number of individuals overall. This reflects the heterogeneity of treatment strategies and the fact that a significant subgroup may benefit from this approach, particularly those with long-term responses to first-line endocrine therapy.

Furthermore, while the full results of the SONIA trial are still awaited, the evolving treatment landscape has already prompted the adoption of second-line CDK4/6 inhibitors in some countries. This is partly due to concerns over cumulative toxicities, both medical and financial, when using CDK4/6 inhibitor universally in the first line. In these contexts, a sequential strategy, reserving CDK4/6 inhibitors for later lines of therapy, remains a relevant and potentially beneficial approach for selected patients. Ongoing trials will provide further clarity on optimal sequencing strategies, but current clinical practice demonstrates that second-line CDK4/6 inhibitors continues to play an important role in managing metastatic breast cancer. 6th and 7th International Consensus Guidelines for the management of advanced breast cancer, informed by the results of the SONIA trial, indicate that using endocrine therapy alone as a first-line treatment is an acceptable option for selected patients—such as those with a low disease burden, a long disease-free interval, specific patient preferences, or challenges with treatment accessibility (32).

Patients with a lower disease burden may exhibit better treatment responses. However, in our cohort, neither oligometastatic disease nor liver metastasis were significant predictors of progression-free survival. This implies that the differential response to treatment may not be solely explained by disease extent, but possibly by underlying biological factors, such as metabolic activity and tumor biology, as indicated by lower baseline SUV values.

These observations could have broader implications for the management of metastatic breast cancer, particularly for incorporating FDG-PET-CT as a biomarker of biological behavior in future treatment strategies. If confirmed in other studies, this would suggest a role for metabolic imaging in guiding the use of therapies, such as targeted treatments. Despite the potential advantages of using PET-CT for monitoring treatment response, several obstacles hinder its routine clinical implementation. One significant barrier is the inconsistent reimbursement for PET scans, which limits their accessibility for regular monitoring. Additionally, the interpretation of metabolic changes, such as *de novo* FDG uptake without corresponding CT abnormalities or ambiguous increases in FDG uptake, poses a challenge for clinical decision-making. Currently, there are no established guidelines that address these scenarios in the CDK4/6 inhibitors treatment, leaving clinicians with limited tools to navigate these complex situations. Furthermore, the expertise required to interpret PET-CT results may vary between institutions, particularly between cancer centers and non-specialized facilities. This variability underscores the need for standardization and validation of PET-based monitoring in metastatic breast cancer. Until such validation is available, conventional imaging modalities may remain the primary tools for treatment monitoring in this population.

Several studies have compared the efficacy of FDG-PET-CT with contrast-enhanced CT (CE-CT) in the context of metastatic breast cancer (19). In a retrospective analysis of 65 patients undergoing various systemic treatments—including chemotherapy, immunotherapy, and endocrine therapy—tumor response as assessed by FDG-PET-CT was found to be a superior predictor of both progression-free survival and disease-specific survival compared to response evaluations using CE-CT (18). Similarly, another study comprising FDG-PET-CT results from 31 patients, treated with different systemic therapies such as endocrine therapy, chemotherapy, and anti-HER2 agents, demonstrated that FDG-PET-CT was more sensitive than CE-CT in monitoring treatment response. In particular, FDG-PET-CT more frequently identified regressive disease, whereas CE-CT tended to report stable disease more often, potentially underestimating treatment efficacy (19). Notably, in a mixed cohort of patients treated with a range of systemic therapies—including endocrine therapy, chemotherapy, trastuzumab, pertuzumab, and CDK4/6 inhibitors—FDG-PET-CT detected disease progression earlier than CE-CT in most cases, with a clinically meaningful delay of up to six months for CE-CT. This early detection by FDG-PET-CT could have significant implications for timely treatment adjustments (20). In another study involving 76 patients with metastatic breast cancer, FDG-PET-CT was shown to be more effective in detecting bone metastases compared to CE-CT, while CE-CT was more sensitive in identifying lung and liver metastases. These findings highlight the complementary role that both imaging modalities might play in comprehensive metastatic disease assessment (21). Despite these promising findings, prospective multicenter studies are still needed to further evaluate key factors such as patient survival, quality of life, and the cost-effectiveness of replacing conventional imaging with FDG-PET-CT. Until these comprehensive studies are conducted, it is premature to draw firm conclusions or make definitive recommendations for FDG-PET-CT in future clinical guidelines (19).

Studies assessing metabolic response (24, 25) require at least two PET scans: one before CDK 4/6 inhibitor initiation and another after some treatment period. This might not be feasible in daily clinical practice due to a monitoring strategy commonly based on contrast-enhanced computed tomography. Our findings suggest that a single PET scan performed before CDK 4/6 inhibitors commencement may suffice as a prognostic tool.

In this study, we used SUVmax as the primary metabolic parameter for analysis, acknowledging its role as a widely accepted and standardized tool in clinical practice. However, it is important to consider the limitations associated with using SUVmax, particularly when dealing with patients who have multiple lesions. The use of SUVmax from the most metabolically active lesion may not fully account for inter- and intra-tumoral heterogeneity, as a single high-uptake lesion could mask the presence of less metabolically active lesions. Alternative metrics such as the SUVmean of all lesions, whole-body metabolic tumor volume (MTV), and total lesion glycolysis (TLG) have been proposed to address this variability and provide a more comprehensive assessment of disease burden. These whole-body metrics take into account the metabolic activity across all lesions, offering a more nuanced understanding of tumor heterogeneity. However, studies that evaluate these parameters in the context of CDK4/6 inhibitor therapy are limited and mostly small-scale (22–24). While such advanced metrics can provide deeper insights, their implementation remains challenging in routine clinical practice due to the complexity and time required to calculate these values, even with the aid of semi-automated tools. SUVmax, on the other hand, is readily available and easier to integrate into standard workflows, making it the preferred metric for daily use. Our study demonstrates that, despite its simplicity, SUVmax retains prognostic value in assessing patient outcomes under CDK4/6 inhibitor therapy.

Another approach in imaging to predict response involves the utilization of radiomic features. When compared to the standard anatomic response evaluation using RECIST 1.1, delta radiomic features have demonstrated the ability to predict a lack of response earlier (33). Interestingly, 16α-^18^F-fluoroestradiol (^18^F-FES), an ER-targeting PET radiotracer, has shown promising results in various situations, such as selecting appropriate patients for endocrine treatment, assessing ER status in lesions that are challenging to biopsy, and solving inconclusive findings from other studies (34). Additionally, liquid biopsy-derived biomarkers could complement imaging approaches (12, 13).

We have to acknowledge the limitations of our study. The main limitation is its retrospective character. Nonetheless, the groups with low SUVmax and high SUVmax were well balanced according to critical clinicopathologic factors affecting survival, including performance status, *de novo* metastatic status, and oligometastatic disease. The inclusion of a relatively small number of patients is another limitation. Remarkably, even with this limited sample, fluorodeoxyglucose positron emission tomography-computed tomography emerged as a robust survival prediction tool.

In our study, we observed a significant difference in PFS between the high and low SUVmax groups, while no statistically significant difference in OS was detected. This discrepancy is not uncommon in clinical research, particularly in studies with limited sample sizes and follow-up durations. PFS is often selected as the primary endpoint in trials because it reflects the time to disease progression or death, offering an earlier measure of treatment efficacy. In contrast, OS requires longer follow-up periods and larger sample sizes to detect differences, as it accounts for multiple factors beyond disease progression, including post-progression therapies and individual patient factors. Moreover, many clinical trials demonstrate significant improvements in PFS without necessarily showing a corresponding OS benefit within the study period. In our case, the median follow-up was likely adequate for detecting differences in PFS, but insufficient to capture the more prolonged effects needed to observe differences in OS. Additionally, the relatively small sample size further limits the statistical power to detect differences in OS. Future studies may provide more definitive conclusions on the prognostic value of SUVmax in overall survival outcomes.

To validate our findings, larger prospective trials are essential. Nonetheless, real-world data like ours might help guide complicated clinical decisions in daily practice while awaiting those studies.

## Conclusions

FDG-PET-CT performed before cyclin-dependent kinase 4/6 inhibitor commencement is a valuable prognostic tool in hormone receptor-positive human epidermal growth factor receptor 2-negative advanced breast cancer. Notably, patients with a maximum standardized uptake value of less than 8.4 experienced extended progression-free survival compared to those with higher SUVmax.

## Data Availability

The raw data supporting the conclusions of this article will be made available by the authors, without undue reservation.

## Supplementary material

Supplementary Figure 1 Receiver Operating Characteristic (ROC) Curve and Area Under the Curve (AUC) for SUVmax.
