# Supplementary figures and images for: The role of FDG PET assessment in patients with advanced breast cancer treated with cyclin-dependent kinase 4/6 inhibitors in the second-line setting
Source: Front Oncol. 2024 Dec 3;14:1454844. doi: 10.3389/fonc.2024.1454844 (PMC11653356; doi:10.3389/fonc.2024.1454844)

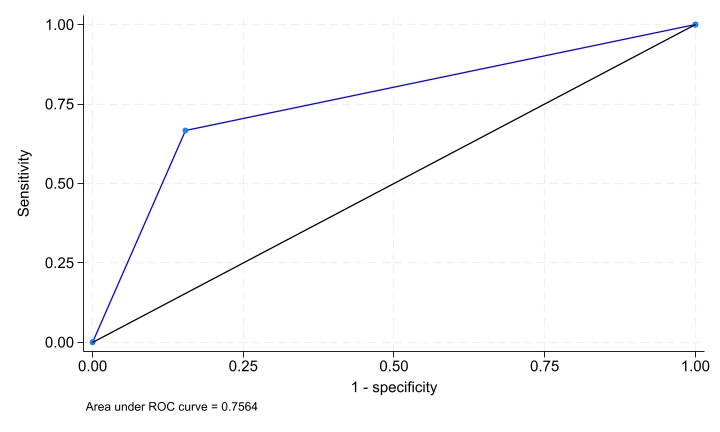

Supplement: Supplementary Figure 1 — Receiver Operating Characteristic (ROC) Curve and Area Under the Curve (AUC) for SUVmax. [file Image1.jpeg]
